# Application of Low-Density Oil Well Cement Slurries Containing Ceramic Microspheres Associated with Sodium Silicate: An Eco-Friendly Alternative

**DOI:** 10.1021/acsomega.5c07197

**Published:** 2025-11-28

**Authors:** João A. N. A. Lima, Luiz E. P. Santiago, Maxwell G. Silva, Cristiane R. Miranda, Renata M. Braga, Júlio C. O. Freitas

**Affiliations:** † Laboratory of Cement, Institute of Chemistry, 28123 Federal University of Rio Grande do Norte (UFRN), 59078-970 Natal, RN, Brazil; ‡ Institute of Chemistry, Federal University of Rio Grande do Norte, 59078-7970 Natal, RN, Brazil

## Abstract

The cement industry is a significant contributor to global pollution, accounting for approximately 7% of the carbon dioxide emissions. This has led to the development of sustainable, high-performance materials for sensitive applications such as oil well cementing. This study developed and characterized a cementitious composite, in which part of the cement was replaced by hollow ceramic microspheres (MS) combined with sodium silicate. This innovative approach resulted in a more environmentally friendly product by reusing low-value industrial waste. Mechanical and chemical characterizations, such as compressive strength, shear stress, pressure analysis, X-ray fluorescence (XRF), X-ray diffraction (XRD), and scanning electron microscopy (SEM), were conducted to evaluate performance. The optimized paste was further analyzed through high-pressure rheology, providing key experimental data for reservoir simulations. The material showed Bingham-type rheological behavior and achieved a compressive strength of 9.35 MPa, which is significantly higher than that of conventional pastes (4 MPa). Additionally, it met the stability standards of the Brazilian oil industry, confirming its suitability for oil wells with low fracture gradients.

## Graphical abstract

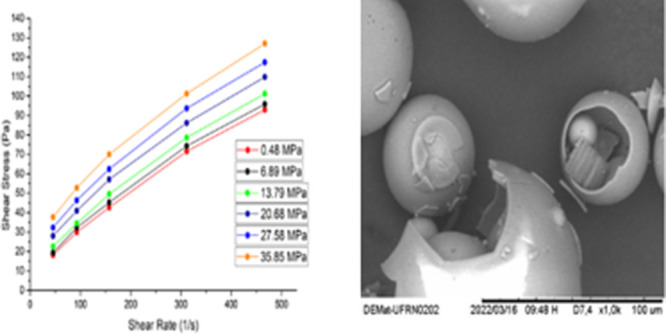

## Introduction

The cement industry is a major pollutant, responsible for approximately 7% of global carbon dioxide emissions.^1^ This scenario has encouraged the search for alternative technologies, particularly the development of environmentally friendly materials capable of partially replacing Portland cement.^2^

The use of common industrial byproducts such as fly ash and ground granulated blast-furnace slag (GGBFS) has been extensively studied, as the pozzolanic activity of these materials improves the mechanical properties of hardened cement pastes.^3,4^ However, although cementitious systems based on these constituents exhibit good mechanical and chemical resistance, they still present serious drawbacks, such as low early strength. This behavior results from the insufficient pozzolonic activity of these materials. Consequently, other types of industrial residues with higher pozzolanic activity have been investigated, such as hollow ceramic microspheres (MS).

MS are classified as a type of fly ash, a byproduct of coal combustion characterized by a hollow spherical shape filled with air. This residue exhibits high pozzolanic activity and, when incorporated into cement pastes, provides several advantages, including reduced paste density, lower greenhouse gas emissions, and improved mechanical and physical properties.^5–7^

A brief timeline of research on the development of new cementitious materials shows that Rita et al.^8^ investigated different compositions of class G cement pastes containing MS to reduce paste density and the hydrostatic column weight during operations. The authors compared the compressive strength and thickening time with those of conventional pastes. The results show that the lighter slurry presented a longer thickening time and higher compressive strength compared to conventional slurries.

The study by Elmrabet et al.^9^ brought new promising aspects related to the use of cements incorporated with fly ash, demonstrating that the pozzolanic properties of fly ash result in higher cement strengths. The optimized mixture consisted of 65% ordinary Portland cement (OPC) and 35% fly ash. In the study by Adjei et al.,^4^ the authors developed a lightweight slurry using fly ash in combination with GGBFS and silica fume. The synergy of these materials provided improved slurry properties, yielding compressive strengths above 40 MPa. Furthermore, the authors obtained a product with desirable rheological behavior, which displayed intrinsic thixotropy, making it suitable for formations with low circulation.

Subsequently, the study by Fantu et al.^10^ investigated the relationship between compressive strength variation and the progressive substitution of cement by fly ash. The results show an increase in the compressive strength with the addition of fly ash. However, substitutions above 10% did not provide further improvements in the slurry performance. The optimal mixture reported by the authors was able to replace 10% of the cement with fly ash, yielding compressive strengths of 67.20 and 64.10 MPa.

In view of the aspects discussed so far, understanding the rheological and mechanical properties enables the development of sustainable, high-performance materials designed for specific and sensitive applications, such as oil well cementing. Therefore, the objective of this study was to develop lightweight cement paste systems using hollow ceramic MS (fly ash) combined with sodium silicate, considering that sodium silicate is a widely used alkaline activator due to its compatibility with cement.

The synergy between these materials resulted in enhanced bonding properties, as well as providing greater adhesion and cohesion capacity among the cement constituents.^11^ This allowed the investigation of how the breakdown of hollow MS alters the typology of lightweight cement slurries as well as the evaluation of the feasibility of the developed material in low-fracture-gradient wells, an essential area of drilling that requires the constant development of improved materials. To this end, an investigation with different slurry compositions was carried out to explain the phenomena involved, employing a methodology capable of simulating various operational conditions to which the lightweight cement slurry would be subjected in the field. This methodology, to the authors’ knowledge, has not yet been investigated in this context, particularly under high-pressure rheology tests.

## Materials and Methods

### Materials

Hollow MS donated by the company Adexim-Comexim (Brazil) had their chemical composition determined by X-ray fluorescence spectroscopy (XRF), as shown in Table 1. The cement used was a Class G cement, supplied by Mizu Cimentos Especiais (Bbrazil). Sodium silicate was purchased from Merck (Brazil). Other materials used in the formulation of the slurries included defoamer DEF-1520 and setting retarder CM-1599, both obtained from AGENA (Brazil). The fluid loss control additive BQ-FLC-30 and the dispersant BQ-FLUX-20 were supplied by BQMIL (Brazil), and bentonite was provided by Bentonit União Nordeste (Brazil).

**Table 1:** Chemical Composition of MS Analyzed by XRF

component	abbreviation	%
silicon dioxide	SiO_2_	53.32
aluminum oxide	Al_2_O_3_	31.49
iron oxide	Fe_2_O_3_	5.01
potassium oxide	K_2_O	4.55
calcium oxide	CaO	1.66
magnesium oxide	MgO	1.41
titanium dioxide	TiO_2_	1.09
sodium oxide	Na_2_O	0.65
phosphorus pentoxide	P_2_O_5_	0.65
sulfur trioxide	SO_3_	0.13
others		0.04

### Cement Slurry Preparation

#### Preparation of Cement Slurries

The specification of cement slurries used in oil wells follows the standards NBR 9831:2020 and API SPEC 10A. With regard to slurries with additives, the procedure followed the practical guidelines outlined in API RP 10B-2. The preparation was carried out using a mixer with time and rotational speed control, manufactured by Chandler Instruments Company (USA), model 30-60. The slurries were homogenized in an atmospheric pressure consistometer, also from Chandler Instruments Company, model 1200. The mixing speed and time were standardized to ensure that the mixing energy is comparable to that used in field equipment.

#### Determination of the Slurry and Microsphere Densities

To determine the final density of the slurries, a pressurized mud balance from RIGCHINA (China), model RPMB-31, was used. Additionally, helium (He) pycnometry was applied to measure the density of the MS. This technique was chosen because He molecules are small enough to penetrate the pores of the material.^12^ For this purpose, an Ultrapyc helium intrusion pycnometer from Anton Paar (Austria) was employed.

#### Compressive Strength of MS

The compressive strength of the MS under pressure was evaluated indirectly by monitoring changes in the slurry density. Due to the low density of the MS, an increase in this parameter indicates particle breakage. The experimental procedure was conducted as follows: The slurry was prepared and placed in the atmospheric pressure consistometer cell. The slurries with densities of 1.32 and 1.44 g/cm^3^ were then subjected to increasing pressures of 1.72, 3.44, 6.89, 13.79, 20.68, and 27.58 MPa for 45 min. After each pressure stage, the slurry density was measured. All tests were performed in triplicate to ensure the accuracy and reproducibility of the experimental data.

### High-Pressure Rheology Tests

The determination of the rheological parameters is crucial for characterizing the properties of cementitious materials. In this study, high-pressure rheology tests were conducted to characterize the slurries by using a pressurized rheometer, model 7500, from Chandler Engineering (USA). During the test, the cement slurry was subjected to a pressure curve ranging from 0.48 to 35.85 MPa. Initially, the slurry was conditioned at 150 rpm until the rheometer reached the test temperature of 52 °C. Measurements began after system stabilization, with the first reading taken at 0.48 MPa. After 30 min from slurry insertion, pressure was incrementally increased to 6.89, 13.79, 20.68, 27.58, and 35.85 MPa, totaling a test duration of 193 min.

Figure 1 visually presents the pressure ramp applied during high-pressure rheology tests.

**Figure 1:**
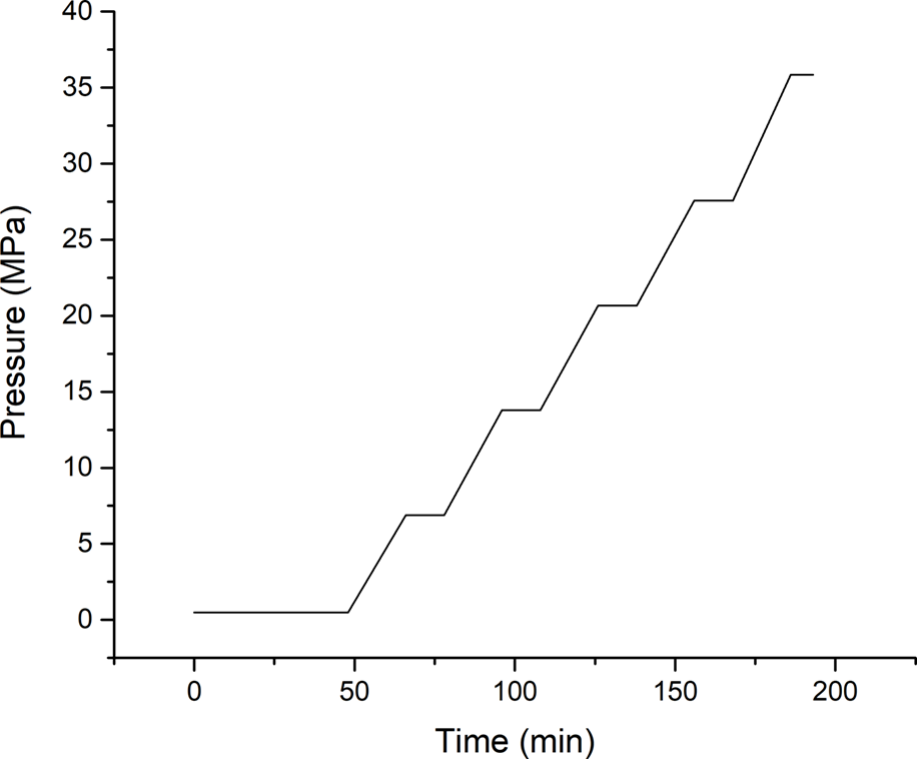
Pressure ramps in high-pressure rheology tests conducted at a temperature of 52 °C.

### Characterization

In order to properly characterize the samples, the following set of techniques was employed:

To determine the average diameter distribution of the MS, laser diffraction particle size analysis by a dry method was performed using a Particle Size Analyzer 1090 Cilas from New Life Scientific (USA).

The phase composition of the samples was analyzed using an X-ray diffractometer (XRD), model D8 Advance, manufactured by Bruker (USA). The tests lasted 60 min, covering a 2θ range from 10° to 70° with a scan step of 0.02°, using a CuKα radiation source. The reference database used for phase identification was the JCPDS card 96-900-0712.

Regarding the microstructure of the cement pastes, field emission scanning electron microscopy (FE-SEM) was performed using an Auriga 40 microscope from Zeiss Evo (Germany).

### Compressive Strength Tests

For the compressive strength tests, the scenario of a well with a depth of 1800 m, without a water column, and with a geothermal gradient of 1.5 °F/100 ft was considered. The mechanical compressive strength tests were performed according to the guidelines established in PROCELAB (2014), starting with the casting of the formulated slurries into three metallic cubic molds with a 50 mm edge length. The distance between opposite faces should be 50.8 ± 0.13 mm, with all angles between the mold walls of 90 ± 0.5°, measured at points slightly away from the face intersections. The molds were then subjected to a thermostatic bath at the static bottom-hole temperature (76 °C) for 24 h. After curing, the specimens were tested in a universal testing machine, model Autograph AG-I (Shimadzu, Japan), with a selected loading capacity of 72 ± 7 kN/min. As the acceptance criterion for the data, a maximum relative deviation of 10% was adopted for each slurry formulation, with each test carried out in triplicate.

### Stability Tests

The objective of the stability tests was to evaluate phase separation effects, such as sedimentation and flocculation, in order to mitigate potential failures in the material. These tests were conducted according to API RP 10B-2. The slurry was subjected to a temperature ramp from ambient conditions to the well’s circulating temperature (48 °C). After this stage, the slurries were placed into cylindrical settling tubes and cured for 24 h in an atmospheric bath preheated to the static bottom hole temperature (76 °C).

After curing, the subsidence of the specimen was assessed. Subsequently, the specimen was sectioned into four equal parts, and the density difference between the top and bottom sections was measured according to eq 1.

$$\Delta \rho =\rho_{\mathrm{top}}-\rho_{\mathrm{bottom}}$$ 1

According to PROCELAB,^13^ the system can be considered stable when the density variation calculated by eq 1 is less than 0.06 g/cm^3^. This value was adopted as the acceptance criterion for the samples in this study.

## Results

### Particle Size Analysis and Mean Particle Diameter Distribution of MS

The particle size was verified based on the technical datasheet provided by the manufacturer, who offers MS with different diameters in their portfolio: 100, 150, and 300 μm, respectively. For the assessment in this section, samples with a diameter of 100 μm supplied by the manufacturer were used.

Figure S1 in the Supporting Information presents the results of the particle size distribution analysis of the MS, showing a uniform particle size distribution. According to the obtained data, the additive presents an average particle diameter of 88.87 μm, distributed as follows: 51.25 μm (10%), 87.18 μm (50%), and 133.46 μm (90%).

### X-ray Diffraction of Hollow Ceramic MS

The X-ray diffraction (XRD) analysis was carried out to identify the phases present in the MS samples. From the major peaks observed in Figure 2, it was possible to confirm that the material corresponds to an aluminum silicate in the form of sillimanite (Al_8_Si_4_O_2_). It should be emphasized that other peaks appearing in Figure 2, which were not labeled, correspond to minor peaks belonging to the same phase. The reference card used for this identification is JCPDS 96-900-0712.

**Figure 2:**
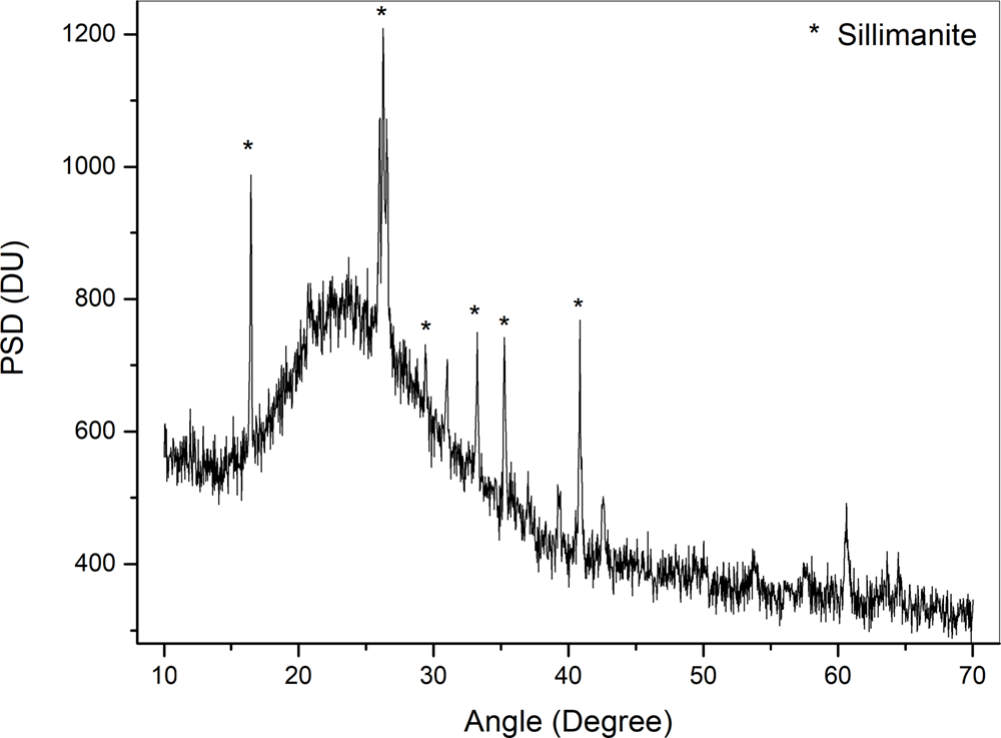
XRD pattern of microspheres.

It is worth highlighting the characteristic amorphous halo of the material, observed in the range between 10° and 30°. This feature emphasizes the pozzolanic activity of the MS. Materials with high pozzolanic activity tend to react with portlandite, thereby accelerating both the hydration rate and the heat of hydration to form a C–S–H gel. Such characteristics enabled the developed paste to present rapid compressive strength development.^14–16^

### Evaluation of the Breakage of Hollow Ceramic MS Under Shear

In order to understand the effect of MS breakage, a preliminary study was conducted to evaluate the effects of shear stress. The specific gravity of the MS was previously measured using helium pycnometry with a determined value of 0.74 g/cm^3^. Based on this, cement slurries were formulated with densities ranging from 1.32 to 1.50 g/cm^3^, maintaining a fixed MS content of 30% in weight.

The samples were subjected to a shear process at 12,000 rpm for 2 min to assess the impact of particle breakage on the slurry density. The detailed description of the slurries before and after the shear tests is provided in Table 2.

**Table 2:** Summary of the Preparation of Pastes for Preliminary Studies, Setting the Substitution of Cement by MS at a Percentage of 30% in Mass^a^

density before break (g/cm^3^)	W/C ratio^b^ (%)	cement (g)	MS (g)	water (g)	antifoam (g)	density after break (g/cm^3^)
1.32	107.16	333.22	99.97	357.07	0.58	1.37
1.38	78.94	395.38	118.61	312.10	0.69	1.41
1.44	58.39	457.53	137.26	267.14	0.80	1.55
1.50	42.75	519.69	155.91	222.18	0.90	NI

a NI: Not Identified.

b Water-cement ratio (%).

After the procedure, an increase in the specific gravity of the MS was observed, from 0.74 to 1.08 g/cm^3^, which resulted in a significant increase in the slurry density, as shown in Table 2. This behavior indicates that when designing lightweight slurries, it is essential to consider the mass fraction of MS and how their breakage affects slurry integrity. Therefore, the processing conditions must be carefully evaluated.

It should be noted that it was not possible to measure the effect of MS breakage under shear in the slurry with a formulation of 1.50 g/cm^3^, due to mixing difficulties at this stage of the experiment, considering that no dispersing additives were used (see Figure S2).

Figures 3 and 4 show the MS in their intact state and after being subjected to shear, respectively. The breakage of the MS can be visually verified in Figure 4 by the red markings.

**Figure 3:**
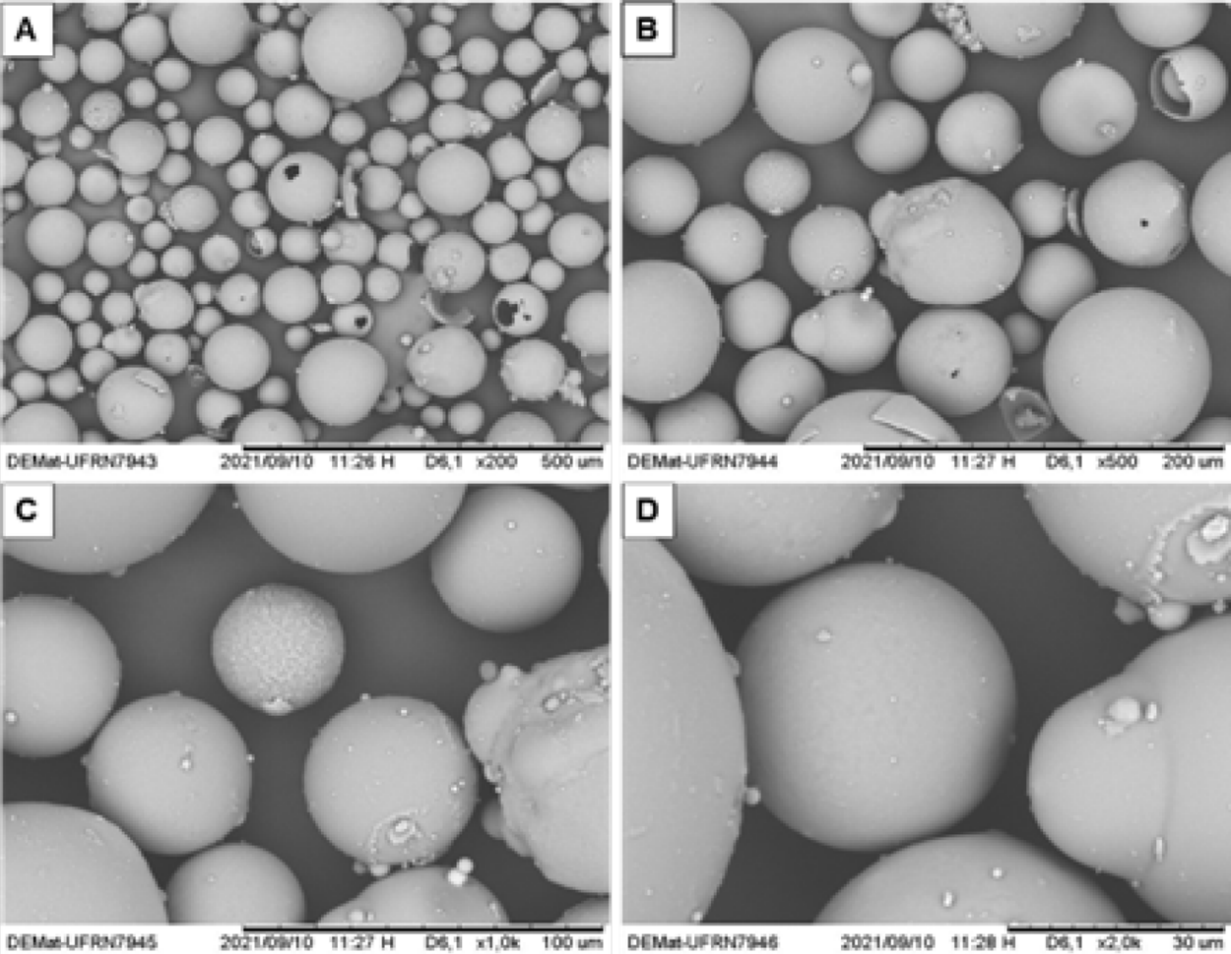
SEM-SE micrographs of intact hollow ceramic microspheres (MS). (A) 200× magnification, (B) 500× magnification, (C) 1000× magnification, and (D) 2000× magnification.

**Figure 4:**
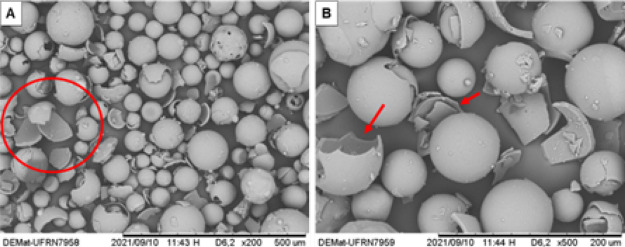
SEM-SE micrographs of hollow ceramic microspheres (MS) after shearing at 12,000 rpm for 2 min. (A) 200× magnification, (B) 500× magnification.

### Evaluation of Ceramic Hollow MS Crushing under Pressure

A crucial factor that profoundly affects the integrity of cement slurries is the influence of the hydrostatic pressure exerted by the wells, especially in deep wells.^17^ Therefore, it is essential to analyze the effect of the pressure on cement slurries synthesized with MS. To this end, a mixture containing only MS and water was previously subjected to a pressure of 33.09 MPa for 45 min to evaluate the density variation of the MS when exposed to high pressures. After the procedure and drying, the density of the MS increased from 0.74 to 1.16 g/cm^3^.

It became evident that the effects of pressure acted more significantly on the MS than the effects of shear stress. Additionally, the morphology of the MS under pressure resulted in the collapse of the spheres, as indicated by the red arrows in Figure 5.

**Figure 5:**
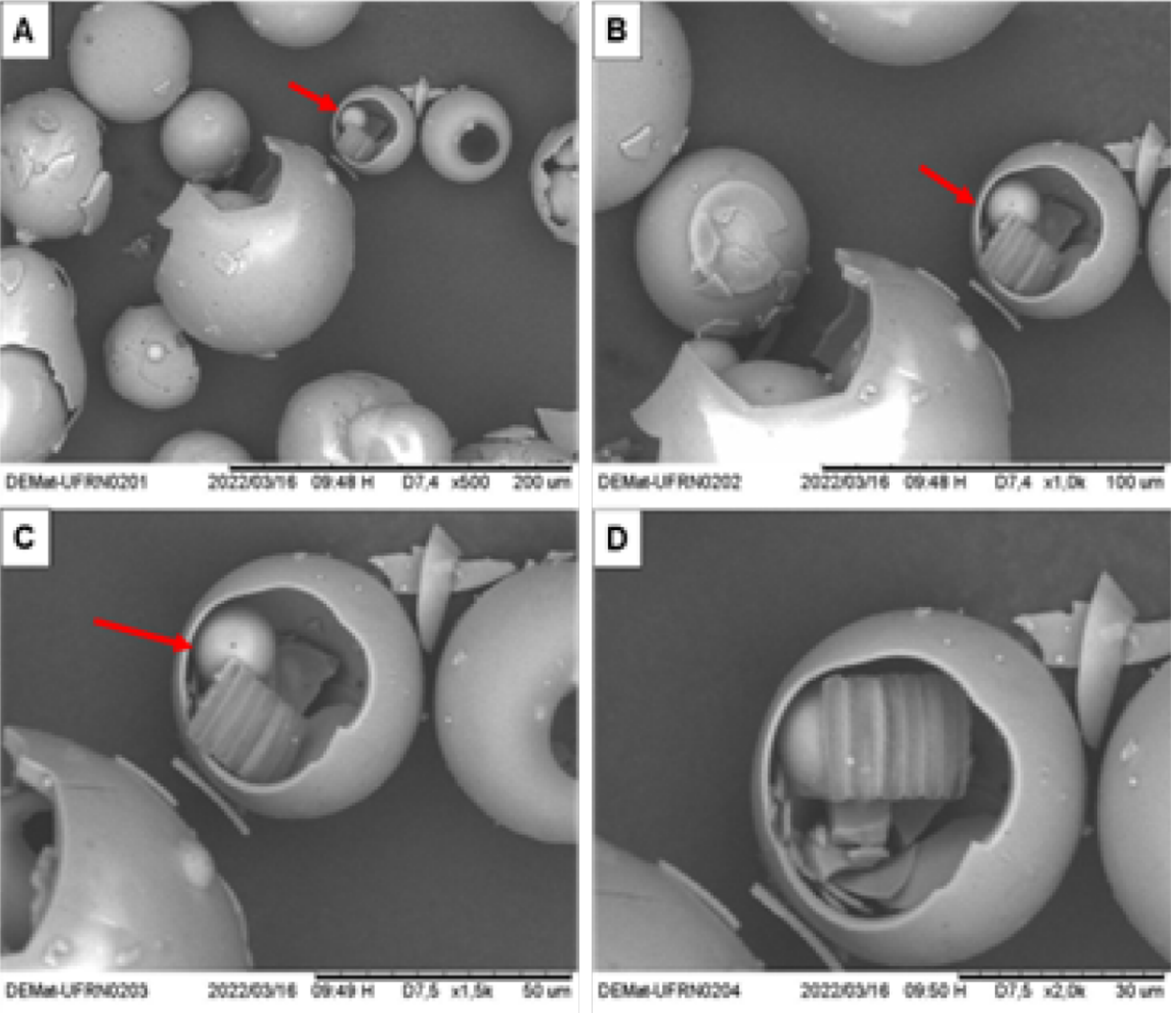
SEM-SE micrographs of hollow ceramic microspheres (MS) after pressure tests of 33.09 MPa for 45 min. (A) 200× magnification, (B) 500× magnification, (C) 1500× magnification, and (D) 2000× magnification.

Subsequently, slurries with densities of 1.32 and 1.44 g/cm^3^, formulated as shown in Table 2, were subjected to different pressure scenarios. The following pressure levels were applied: 1.72, 3.45, 6.89, 13.79, 20.68, 27.58, and 31.03 MPa. It is important to emphasize that for each pressure condition, a new mixture was prepared, and only then was the slurry density measured. The results were extrapolated to create the trend curve shown in Figure 6.

**Figure 6:**
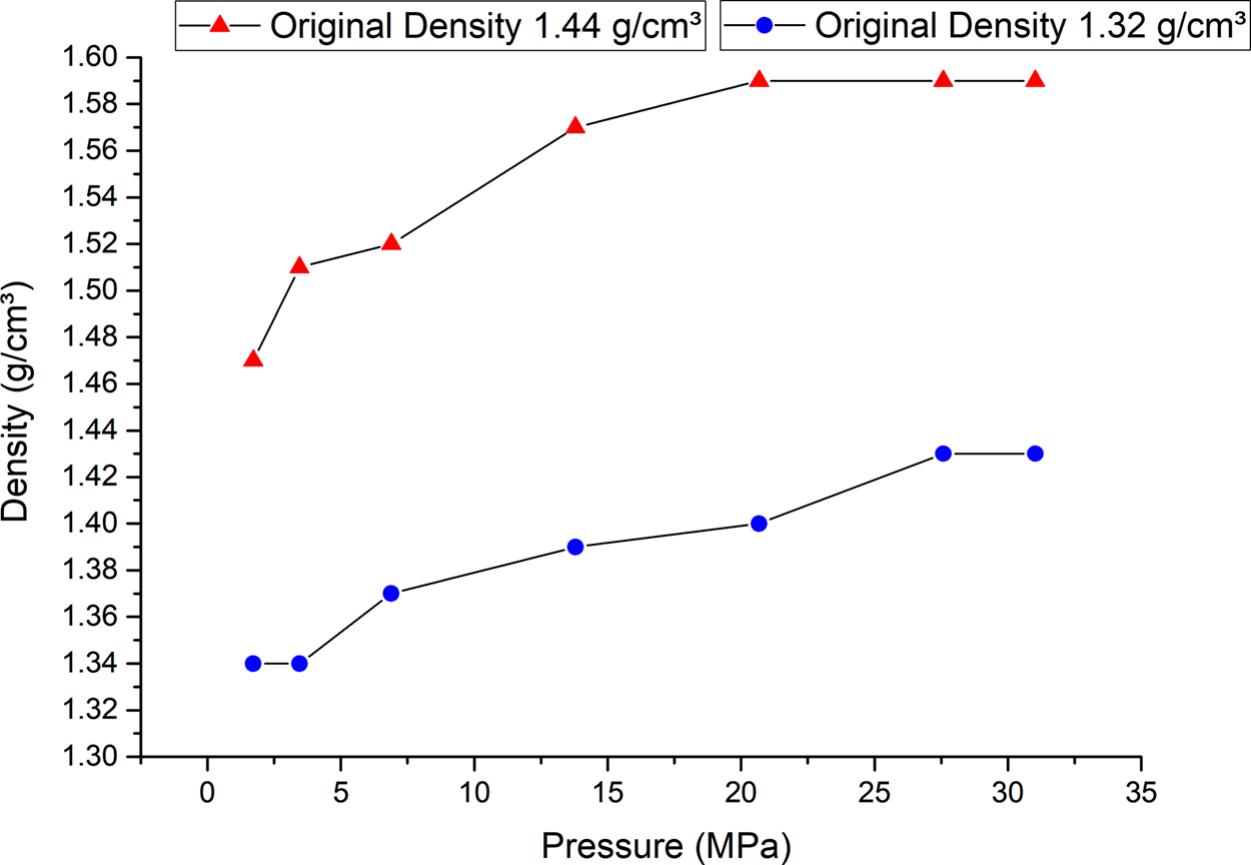
Behavioral changes in the density of two different light pastes with pressure.

From Figure 6, it is clear that the slurry density increases with the applied pressure. However, an asymptotic trend becomes evident from 27.58 MPa onward. From this point, the slurry density no longer showed significant variations, indicating complete crushing of the MS present. Consequently, pressures above this threshold no longer induced changes in the cement slurry density.

### Statistical Analysis of the Relationship between Mechanical Properties and Different Slurry Formulations

Lightweight cement systems, due to their lower water content, tend to lose stability.^16^ In this context, the partial replacement of cement with MS further reduced the water content and generated defective material that failed under relatively low pressures (31 MPa). Considering that the substitution/insertion of materials in the slurry alters the hydration rates,^18,19^ it was necessary to add sodium silicate in an appropriate amount, since the addition of sodium silicate stimulates a “secondary hydration” of cement, resulting not only in the improvement of mechanical properties,^20,21^ but also in the adhesion and cohesion of the slurry materials.

In order to balance these factors, a factorial design was implemented to cover the mentioned premises. Thus, the range of values for each factor was defined based on preliminary tests, ensuring particle cohesion within the system and avoiding secondary issues such as flocculation or sedimentation. In this way, the experimental design involved the determination of the compressive strength of 11 cement slurries, divided into tests with 4 axial points and 3 repetitions at the central point. Furthermore, an analysis of variance (ANOVA) was carried out with a 95% confidence interval.

The polynomial equation that describes the compressive strength is given by eq 2.^22^

$$Y=b_{0}+\sum_{i=1}^{n}b_{i}X_{i}^{2}+\sum_{i=1}^{n}b_{ii}X_{i}^{2}+\sum_{i=1}^{n}\sum_{j=1}^{n}b_{ij}X_{i}X_{j}$$ 2

where *Y* represents the compressive strength, while *b*_0_, *b*_*i*_, *b*_*ii*_, and *b*_*ij*_ are the regression coefficients. The variables *X*_*i*_ and *X*_*j*_ correspond to the decoded parameters. Table 3 presents a detailed description of the masses used in the preparation of the cement slurry formulations.

**Table 3:** Detailed Description of Paste Formulations in Experimental Design

sample	cement (g)	MS (g)	silicate (g)	DEF 1520 (g)	BQ flux (g)	BQ FLC 30 (g)	water (g)
1-B	369.25	45.05	14.27	0.64	1.13	2.22	405.01
2-B	391.39	89.24	15.12	0.68	1.20	2.35	337.59
3-B	446.86	54.52	17.27	0.77	1.36	2.66	368.91
4-B	473.65	107.99	18.30	0.82	1.45	2.84	282.83
5-B	362.88	63.50	14.02	0.63	1.11	2.18	382.45
6-B	476.98	83.47	18.43	0.82	1.46	2.86	314.64
7-B	403.32	40.33	15.58	0.70	1.23	2.42	399.14
8-B	437.97	109.49	16.92	0.76	1.34	2.63	293.61
9-B	419.93	73.49	16.23	0.73	1.28	2.52	348.55
10-B	419.93	73.49	16.23	0.73	1.28	2.52	348.55
11-B	419.93	73.49	16.23	0.73	1.28	2.52	348.55

Figure 7 shows the compressive strength test results for the 11 cement slurry formulations. It should be highlighted that, for comparison purposes, the compressive strength of a conventional lightweight slurry commonly used in low-fracture-gradient fields was also included. This reference slurry has a specific gravity of 1.50 g/cm^3^ and contains 4% BWOC of bentonite, being highlighted in blue in Figure 7.

**Figure 7:**
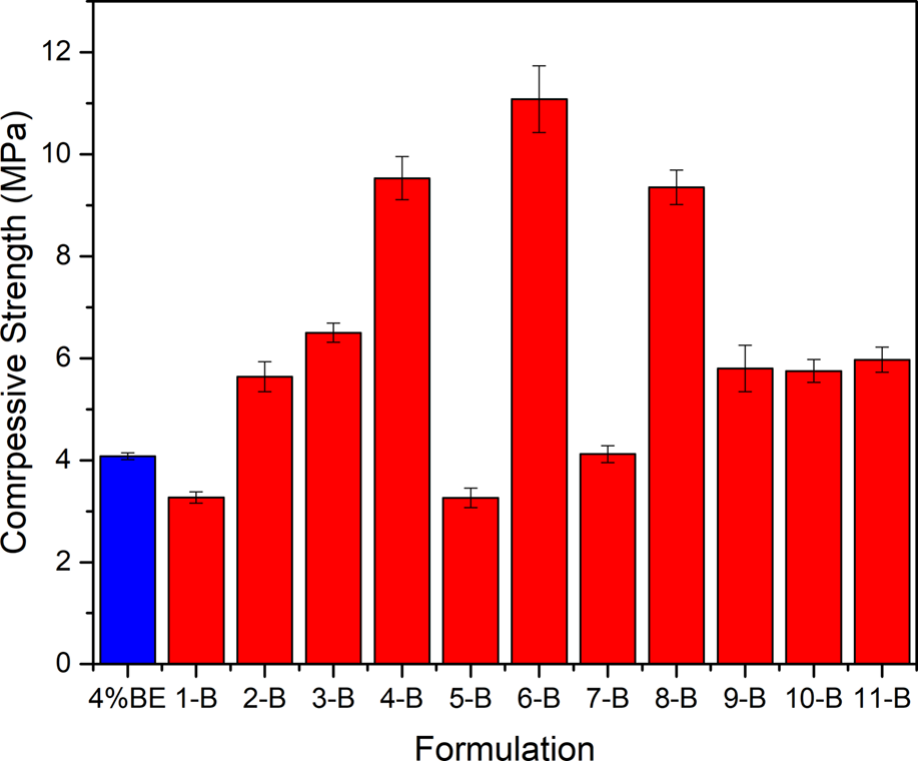
Compressive strength responses of lightweight cement pastes from experimental design, in 1 d, at a temperature of 76 °C.

From Figure 7, the reproducibility of the tests becomes evident, since formulations 9-B, 10-B, and 11-B correspond to three replicates of the central point, showing an average of 5.84 MPa and a standard deviation of 0.009. Another interesting behavior can be inferred from the water-to-cement ratios of the formulations: as this ratio decreases, a higher compressive strength is obtained. This behavior is probably associated with the increased formation of C–S–H gel due to the higher amounts of cement, sodium silicate, and MS present in the slurry formulations.

In addition, a quadratic model adjustment was tested by using ANOVA, ensuring the assessment of variability and model reliability. The principle of ANOVA is to partition the results into two components: one related to model variation and the other to experimental error variation. The statistical results of the ANOVA are listed in Table 4.

**Table 4:** Results of ANOVA for Responses

	sum of squares	degrees of freedom	mean square	*F*_cal_	*F*_cal_/*F*_tab_
regression	63.74	5	12.75		
residual	3.38	5	0.68	18.84	3.73
lack of fit	3.36	3	1.12		
pure error	0.026	2	0.01	86.62	4.53
total	67.13	10	6.71		

The confidence level was set at 95%, allowing for examination of the significance level of the model adjustment. Regarding data quality, the coefficient of determination (*R* ^2^) was 0.9496, meaning that the statistical model derived from the experimental data was able to explain 94.96% of the variance. Furthermore, an *F*-test was performed to assess the statistical significance of the model, which can be considered significant (*F*_cal_/*F*_tab_ > 1). However, when the *F*-test was performed to evaluate the predictive capability of the model, it became clear that the model cannot be considered predictive.

### High-Pressure Rheology Test and Analysis of the Developed Slurry’s Potential

The synthesized lightweight slurries exhibit complex correlations, making it necessary to optimize appropriate proportions of MS and sodium silicate to balance the rheological properties (such as yield stress and viscosity) with the desired mechanical properties (such as compressive strength and durability). Although the addition of MS and sodium silicate can initially improve several characteristics, such as increasing compressive strength, reducing viscosity, and enhancing system fluidity, a trade-off also occurs: the acceleration of hydration reactions, which results in shorter setting times.^21,23^

Based on the slurry formulations from the experimental design, an optimal point was selected for conducting high-pressure rheology tests. To this end, formulation 8-B was chosen due to a set of attractive characteristics, such as the reduced amount of water and cement, combined with appropriate proportions of ME and sodium silicate, which resulted in a cohesive slurry with a compressive strength of 9.35 MPa. This value is higher than the average compressive strength measured for conventional bentonite-based slurries, which reached 4 MPa (Figure 7). The selected formulation yielded a cement slurry with a density of 1.44 g/cm^3^, meeting the stability criterion with a measured stability value of 0.28.

Based on the formulation aspects discussed above, experiments under various shear and pressure conditions were carried out using high-pressure rheology to understand the synergy of these combined effects on the cementitious material. The experimental conditions and results are visually presented in Figure 8.

**Figure 8:**
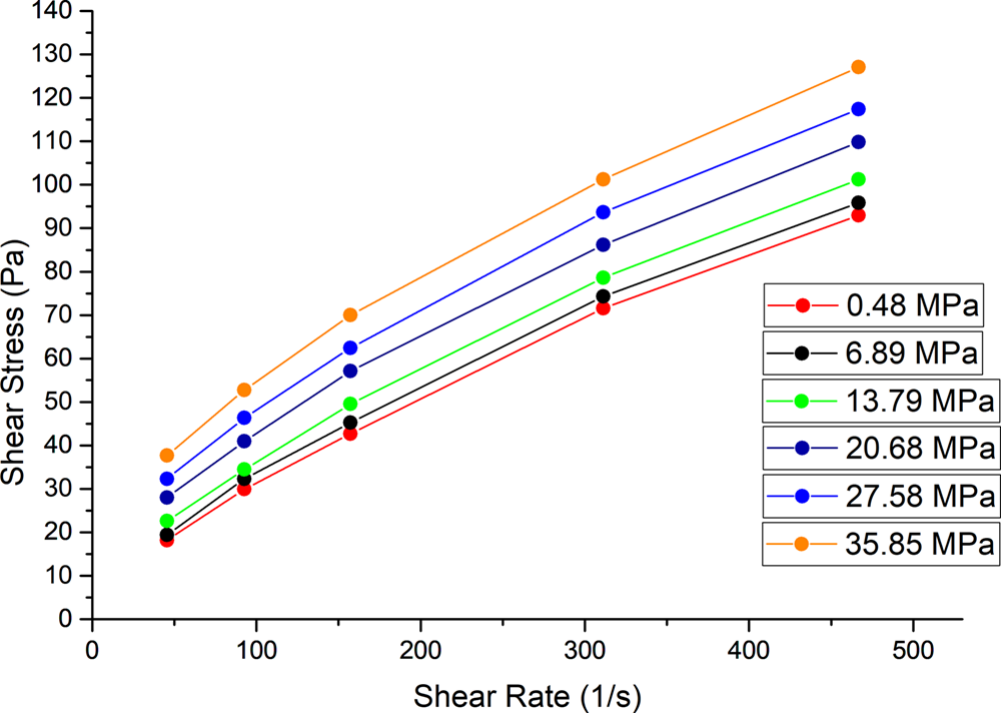
Results from high-pressure rheology tests using the optimized paste-8B.

As seen in previous sections, the increase in shear and pressure of the system promotes the rupture of MS, thereby increasing the slurry density. These two factors directly influenced the rheological parameters of the slurry, as the MS, once broken, lose their “rolling” effect and consequently generate higher resistance during the slurry displacement to the target zone.^24,25^

The most common rheological models capable of explaining the relationship between shear stress and shear rate in lightweight slurries are the Bingham plastic model and the exponential model.^26^ Based on the data from Figure 8, the rheology was classified as Bingham plastic, whose phenomenological representation is expressed by eq 3. In this model, once a yield stress is exceeded, the fluid exhibits a linear relationship between shear stress and shear rate, similar to a Newtonian fluid.^27^

τ=LE+VP×γ 3

where τ is the shear stress in Pa, γ is the shear rate in s^–1^, YS is the yield stress in Pa, and PV is the plastic viscosity in Pa s. The rheological parameters for each pressure level are presented in Table 5, where the regression coefficients demonstrate the good fit of the model to the experimental data.

**Table 5:** Rheological Parameters of the Synthesized Cementitious Material

pressure (MPa)	0.48	6.89	13.79	20.68	27.58	35.85
*R* ^2^	0.99	0.99	0.99	0.99	0.99	0.99
PV (Pa s)	0.16	0.16	0.17	0.17	0.18	0.19
YS (Pa)	15.20	16.02	19.64	23.24	27.91	32.46
*G*_i_ (Pa)	3.90	4.70	6.40	9.10	14.00	20.00
*G*_f_ (Pa)	7.90	10.30	15.10	26.50	42.10	58.00

From the data in Table 5, it was possible to verify that the inclusion of MS and sodium silicate in the lightweight cement slurries produced rheological behavior analogous to that found in studies involving nanomaterials. In such studies, the Bingham plastic model was the most suitable to explain the results.^16,28^ Rheologies with this kind of behavior are more suitable for field applications since they behave like Newtonian fluids after overcoming the yield stress YS.

Furthermore, it is observed that the initial gel strength (*G*_i_), final gel strength (*G*_f_), and yield stress all increased with pressure. This indicates that the increase in these parameters is intrinsically related to the rupture of MS and, consequently, the increase in resistance to slurry displacement.

Unlike other studies that used silica in the synthesized material and focused on improving compressive strength, this study synthesized a lightweight slurry that balanced the use of pozzolanic materials, requiring less water in the formulation while achieving higher compressive strength and meeting technical requirements for application in petroleum wells with low fracture gradient.

## Conclusion

The present study evaluated and synthesized a cementitious material in which cement was partially replaced by hollow ceramic MS in combination with sodium silicate, aiming to generate a more environmentally friendly product. This approach enables the use of a low-value industrial byproduct while remaining applicable to oil wells with low fracture gradients, which are known for their operational challenges. Since the partial replacement of cement using only MS produced a defective material that failed under relatively low pressures (31 MPa), it was necessary to incorporate sodium silicate, an additive that interacts effectively with highly pozzolanic materials such as MS. The results from the high-pressure rheology tests showed that the synthesized lightweight slurry exhibits rheological behavior similar to that of Bingham plastics. Moreover, good operational conditions must be taken into consideration, since pressure and shear effects can lead to the rupture of the MS, altering the cementitious slurry properties and eliminating the rolling effect derived from the incorporation of this material into the slurry.

Even so, the results were promising, demonstrating the potential for replacing conventional cement used in petroleum wells with this new product. Lightweight slurries with specific gravities ranging from 1.32 to 1.50 g/cm^3^ were formulated and subjected to various physical and chemical characterization tests. The optimized lightweight slurry exhibited a compressive strength of 9.35 MPa, a value significantly higher than that of the conventional slurry with a strength of 4 MPa. Finally, further studies are still required to evaluate the reactivity of MS with the slurry, as well as investigations on the dynamics of longer curing times, the role of static sedimentation, and the economic feasibility of using special cements manufactured from the synthesized material on a large scale.

## Supplementary Material



## Notes

Particle size distribution obtained by laser granulometry and image of lightweight cement slurry with 1.5 g/cm^3^ density after shear test at 12,000 rpm for 2 min (PDF)
