# Supplementary material for: Application of Low-Density Oil Well Cement Slurries Containing Ceramic Microspheres Associated with Sodium Silicate: An Eco-Friendly Alternative
Source: ACS Omega. 2025 Nov 28;10(48):58779–87. doi: 10.1021/acsomega.5c07197 (PMC12771115; doi:10.1021/acsomega.5c07197)
Supplement: Supplementary file 1 [file ao5c07197_si_001.pdf]

Application of low-density oil well cement slurries containing ceramic microspheres associated with sodium silicate: an eco-friendly alternative

João A. N. A. Lima [a], Luiz E. P. Santiago [b], Maxwell G. Silva [b,*], Cristiane R. de Miranda [a]; R. M. Braga [b], Júlio C. de O. Freitas [b]

[a]Laboratory of Cement, Institute of Chemistry, Federal University of Rio Grande do Norte (UFRN), 59078-970, Natal-RN, Brazil.

[b]Institute of Chemistry, Federal University of Rio Grande do Norte (UFRN), 59078-970, Natal-RN, Brazil.

* Corresponding author.

SUPPLEMENTARY INFORMATION

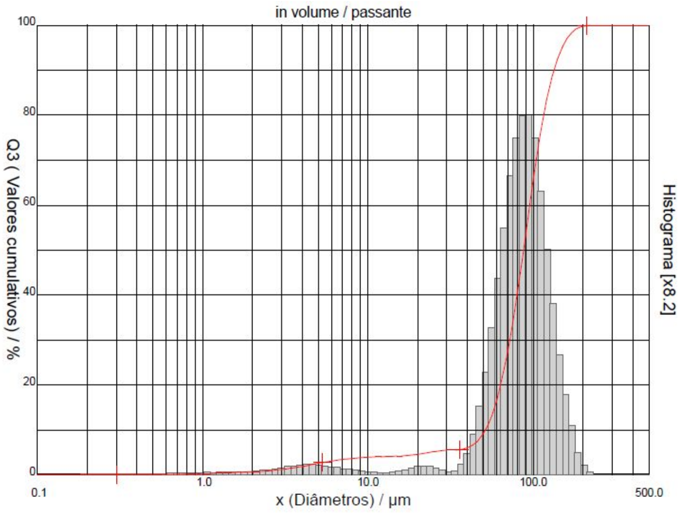

Figure S1. Particle distribution via laser granulometry

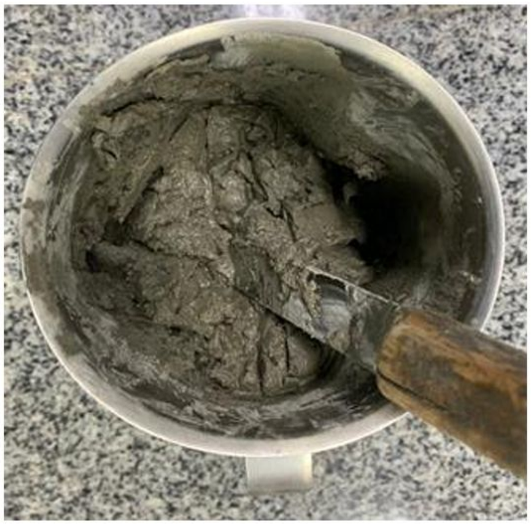

Figure S2. Lightweight cement slurry with 1.5 g/cm³ density after shear test at 12,000 rpm for 2 minutes
